# HIV-1 Tat protein enhances the intracellular growth of *Leishmania amazonensis* via the ds-RNA induced protein PKR

**DOI:** 10.1038/srep16777

**Published:** 2015-11-26

**Authors:** Áislan de Carvalho Vivarini, Renata de Meirelles Santos Pereira, Victor Barreto-de-Souza, Jairo Ramos Temerozo, Deivid C. Soares, Elvira M. Saraiva, Alessandra Mattos Saliba, Dumith Chequer Bou-Habib, Ulisses Gazos Lopes

**Affiliations:** 1 Laboratório de Parasitologia Molecular, Instituto de Biofísica Carlos Chagas Filho, Centro de Ciências da Saúde, Universidade Federal do Rio Janeiro, Rio de Janeiro, Rio de Janeiro, Brazil; 2 Laboratório de Imunobiologia de Leishmanioses, Instituto de Microbiologia Paulo Góes, Universidade Federal do Rio Janeiro, Rio de Janeiro, Rio de Janeiro, Brazil; 3 Laboratório de Pesquisas sobre o Timo, Instituto Oswaldo Cruz, Fundação Oswaldo Cruz, Rio de Janeiro, Rio de Janeiro, Brazil; 4 Departamento de Microbiologia, Faculdade de Ciências Médicas, Universidade do Estado do Rio de Janeiro, Rio de Janeiro, Brazil

## Abstract

HIV-1 co-infection with human parasitic diseases is a growing public health problem worldwide. *Leishmania* parasites infect and replicate inside macrophages, thereby subverting host signaling pathways, including the response mediated by PKR. The HIV-1 Tat protein interacts with PKR and plays a pivotal role in HIV-1 replication. This study shows that Tat increases both the expression and activation of PKR in *Leishmania*-infected macrophages. Importantly, the positive effect of Tat addition on parasite growth was dependent on PKR signaling, as demonstrated in PKR-deficient macrophages or macrophages treated with the PKR inhibitor. The effect of HIV-1 Tat on parasite growth was prevented when the supernatant of HIV-1-infected macrophages was treated with neutralizing anti-HIV-1 Tat prior to *Leishmania* infection. The addition of HIV-1 Tat to *Leishmania-*infected macrophages led to inhibition of iNOS expression, modulation of NF-kB activation and enhancement of IL-10 expression. Accordingly, the expression of a Tat construct containing mutations in the basic region (49–57aa), which is responsible for the interaction with PKR, favored neither parasite growth nor IL-10 expression in infected macrophages. In summary, we show that Tat enhances *Leishmania* growth through PKR signaling.

Diseases caused by the protozoan parasite *Leishmania* spp. can be impacted by human immunodeficiency virus (HIV)-1 co-infection, particularly in countries with a high incidence of both pathogens^1^,^2^. *Leishmania* spp. is one of the most common parasites worldwide and causes approximately 0.2 to 0.4 million episodes of visceral leishmaniasis and 0.9 to 1.2 million cases of cutaneous leishmaniasis each year^3^. Episodes of *Leishmania*/HIV-1 coinfection have been reported in tropical and Mediterranean countries and are frequently associated with a poor clinical prognosis for both diseases^4^,^5^. *Leishmania* modulates cellular signaling pathways, including increasing the expression and activation of the double-stranded RNA (dsRNA)-activated protein kinase R (PKR)^6^. PKR is notorious for its pivotal role in the antiviral response via the expression of type I interferons^7^,^8^. Recently, our group and others reported a novel role for PKR-mediated signaling in the outcome of infections with intracellular protozoan parasites such as *Leishmania* and *Toxoplasma gondii*^6^,^9^,^10^. Depending on the *Leishmania* species involved in the infection, PKR activation exacerbates the intracellular growth of the parasite due to the production of interleukin (IL)-10 and interferon (IFN)-β^10^,^11^.

PKR activity can be regulated by the HIV-1 Tat protein, which is responsible for the transactivation of the HIV-LTR through binding to the trans-activation response element (TAR) in nascent viral transcripts^12^. The Tat protein is released by HIV-1-infected cells, is abundant in the serum and tissues of infected individuals and plays several modulatory roles in addition to regulating HIV-1 replication. Once in the extracellular milieu, HIV-1 Tat interacts with host cells and modulates signaling pathways by binding to external membrane receptors, such as TLR4^13^. The Tat protein can be internalized by different cells including macrophages, and interacts with intracellular signaling kinases, such as PKR^14^. The PKR-Tat interaction can culminate in the activation of the transcription factor NF-κB and the expression of cytokines such as TNF-α and IL-10 15. Additionally, Tat interacts with and is phosphorylated by PKR^16^,^17^, whose kinase activity on Tat increases HIV-1 long terminal repeat (LTR) transcription activity, thereby up-modulating viral production^18^. In addition, NF-κB activation by Tat relies on PKR kinase activity^19^, and so the Tat-induced expression of IL-10 20. Therefore, it is plausible to propose that HIV-1 infection and/or the HIV-1 Tat protein can interfere with intracellular *Leishmania* growth via PKR activity. We previously demonstrated that the exogenous addition of Tat increases the infection load of intracellular *Leishmania amazonensis* in macrophages^21^, and in the present work we investigated the role of PKR in the aggravation of *Leishmania* infection driven by Tat. Our data implicate PKR as a key molecule bridging the effect of the HIV-1 protein on parasite infection.

## Material and Methods

### Reagents

Phorbol-12 myristate-13 acetate (PMA) was purchased from Sigma-Aldrich (St. Louis, MO, USA) and PKR-inhibitor CAS 608512-97-6 was purchased from Millipore (Darmstadt, Germany). The HIV-1 Tat protein was obtained from Dr. J. Brady (National Cancer Institute, National Institutes of Health) and the rabbit antiserum to HIV-1 Tat was obtained from B. Cullen (Duke University Medical Center) through the AIDS Research and Reference Reagent Program (Division of AIDS, National Institute of Allergy and Infectious Diseases, N.I.H.). The lyophilized 86-aa Tat was reconstituted in PBS containing 0.1 mmol/L dithiothreitol (DTT) (Invitrogen, Carlsbad, CA, USA) and 1 mg/mL bovine serum albumin (Promega Corp., Madison, WI, USA). Oxidized Tat was prepared as previously described^21^.

### Cell lines and culture

The human monocytic leukemia cell line THP-1 (ATCC:TIB202TM) was maintained in DMEM medium with high glucose (Vitrocell Embriolife, Campinas, SP, Brazil) supplemented with 10% heat-inactivated fetal bovine serum (Sigma-Aldrich, St. Louis, MO, USA). THP-1 cells were differentiated into macrophages by incubating with 40 ng/mL of PMA for 3 days. Then, the cells were washed 3 times with PBS and incubated with fresh medium for an additional 3 days. RAW 264.7 cells expressing either empty vector (RAW-WT-Bla cells) or a dominant-negative PKR K296R (RAW-DN-PKR cells) were provided by Dr. Aristóbolo Silva (Federal University of Minas Gerais, Brazil). Monocyte-derived macrophages were obtained from peripheral blood mononuclear cells (PBMCs) isolated from buffy coat preparations of human healthy blood donors as previously described^21^.Thioglycolate-elicited peritoneal macrophages from wild-type or PKR-knockout 129 Sv/Ev mice were obtained by injecting 8 mL of serum-free DMEM into the peritoneal cavity. After five days, the cells were washed in PBS one time and then plated in in DMEM medium supplemented with 10% FBS on glass coverslips at a density of 2 × 10^5^/well in 24-well polystyrene plates for subsequent *Leishmania* infection assays. All experimental procedures involving human cells were approved by the Oswaldo Cruz Foundation/Fiocruz Research Ethics Committee (Rio de Janeiro, RJ, Brazil), under the number 397-07. The experimental protocols using mice were approved by the Federal University of Rio de Janeiro Committee for Animal Use (permit numbers: IMPPG 024 and IBCCF171.

### Parasites, culture conditions and infection

*Leishmania (L.) amazonensis* (WHOM/BR/75/Josefa) was used in this study. Promastigote forms were grown at 26 °C in Schneider’s Insect Medium (Sigma-Aldrich) with 10% fetal bovine serum and were used at the stationary growth phase (5–6 days). Macrophages were infected with *Leishmania* promastigotes with a parasite: cell ratio of 5:1 at 37 °C. In some experiments, THP-1 cells were treated with 300 nM of the PKR inhibitor. Recombinant HIV-1 Tat (100 ng/mL) was added to the cell cultures after *Leishmania* infection (the use of this concentration was based on our previous published work^21^). Infected macrophages were counted by light microscopy to assess the infection index, which was calculated by multiplying the percentage of infected macrophages by the average number of parasites per macrophage in Giemsa-stained slides. Promastigote production derived from infected cells was evaluated as follows: after 3 days of infection, non-adherent cells were removed and the adherent cells were washed 3 times with 1x PBS. The adherent cells were added to the wells in 0.5 mL of Schneider medium containing 20% FBS. After five to seven days at 26 °C, the growth of extracellular motile promastigotes originating from the infected macrophages was assessed by counting in a Neubauer chamber.

### Safety of *Leishmania* treatment with Tat and PKR inhibitor

*Leishmania* promastigotes were treated with Tat or oxidized-Tat (both at 100 ng/mL), or with 300 nM of the PKR-inhibitor for 1 h. Then, the promastigotes were washed with PBS and incubated with peritoneal macrophages (obtained as described above) at a 1:5 parasite: cell ratio for 1 h or 48 h at 35 °C in 5% CO_2._ The infection index was calculated as described above and used to determine parasite survival.

### HIV-1 isolate and co-infection

THP-1 cells were infected with the monocytotropic CCR5-dependent HIV-1 isolate Ba-L using viral suspensions containing 5–10 ng/mL of HIV-1 p24 antigen. Excess virus was washed out after 24 hours and then fresh medium was added back. The cells were maintained under standard culture conditions for 10 days. Subsequently, macrophages were infected with *L. amazonensis* promastigotes with a parasite: cell ratio of 5:1 at 37 °C overnight. Non-internalized promastigotes were washed out, fresh medium was added, and the cultures were maintained at 37 °C in 5% CO2 for an additional 72 hours for counting by light microscopy to estimate the infection index. For HIV-1 Tat neutralization, rabbit anti-HIV-1 Tat or the IgG isotype control were employed as follows: the supernatant of HIV-1 infected macrophages was removed and treated with anti-Tat, the IGg1 isotype or iPKR. Fresh medium was added and the promastigotes were allowed to interact with the cultures for one hour. After this period of time, the treated supernatant was added to the infected macrophages and the infection index was estimated as described above.

### Nitric Oxide Production

The Griess reaction was employed to analyze the nitrite (NO2^−^) content as an indicator of NO production in the supernatant of RAW 264.7 cell lines accordingly the manufacturer’s instructions (Sigma-Aldrich, St. Louis, MO, USA). The reaction was read at 540 nm and the NO2- concentration was determined with reference to a standard curve generated using sodium nitrite. The results were expressed as micromolar concentrations of nitrite.

### Immunoblotting

THP-1 cells (1 × 10^6^ cells) were washed twice with ice-cold PBS and then lysed in 100 μl of lysis buffer (50 mM Tris-HCl, pH 7.5, 5 mM EDTA, 10 mM EGTA, 50 mM NaF, 20 mM β-glycerophosphate, 250 mM NaCl, 0.1% Triton X-100, 1 μg/ml BSA and a 1:100 dilution of protease inhibitor cocktail (Sigma-Aldrich, St. Louis, MO, USA) for total protein extraction. For nuclear protein extraction, after infection and/or treatment, the cells were washed twice with 1X PBS and then lysed with 100 μL of buffer A (HEPES 10 mM pH 7.9, 10 mM KCl, 0.1 mM EDTA, 0.1 mM EGTA, 0.25% NP-40 (v/v), and a protease inhibitor cocktail for 10 minutes on ice. Then, the lysed cells were centrifuged for 14.000 g for 1 minute at 4 °C and the pellet was resuspended in 60 μL of buffer C (20 mM HEPES pH 7.9, 0.4 M NaCl, 1 mM EDTA, 1 mM EGTA, 20% glycerol, and protease inhibitor cocktail) and incubated on ice for 20 minutes. The lysed cells were centrifuged at 14.000 g for 5 minutes, and the supernatant containing nuclear proteins was collected in a new tube. Protein extracts were subjected to electrophoresis in 10% SDS-polyacrylamide gels and transferred to a nitrocellulose membrane (Amersham Biosciences, Piscataway, NJ, USA). After blocking with 5% nonfat dry milk in TBS with 0.1% Tween-20 (TBS-T), the blots were incubated overnight with antibodies against PKR, phospho-eIF2α Ser51, α-Tubulin, GAPDH, Lamin A/C (Cell Signaling Technology, Danvers, MA, USA), phospho-PKR Th451 (Millipore, Darmstadt, Germany), NFκB-p65, iNOS or β-actin (Santa Cruz Biotechnology, Dallas, TX, U.S.A), followed by anti-rabbit or anti-mouse horseradish peroxidase-conjugate IgG (1:4000). Then, the membranes were washed 3 times with 0.1% TBS-T after each incubation and the proteins were detected by the ECL chemiluminescent detection system (Amersham Biosciences).

### Quantitative Real Time RT-PCR

Total RNA from THP-1, wild-type RAW 264.7 and DN-PKR cells (1 × 10^6^ cells) was extracted with the Invitrap® Spin Cell RNA mini kit (STRACTEC Molecular GmbH, Berlin, Germany). A 1 μg aliquot was reverse transcribed to first-strand cDNA with ImProm-II (Promega) and the oligo(dT) primer according to the manufacturer’s instructions. The DNA sequences of the primers used in this work are: Hu-PKR-F: 5’-GACCTTCCTGACATGAAAGAA-3′ and Hu-PKR-R: 5′-AACTATTTTTGCTGTTCTCAGG-3′; GAPDH-F: 5′-TGCACCACCAACTGCTTAGC-3′ and GAPDH-R: 5′-GGCATGGACTGTGGTCATGAG-3′; Mu-iNOS-F: 5′-CAGCTGGGCTGTACAAACCTT- 3′ and Mu-iNOS-R: 5′-CATTGGAAGTGAAGCGTTTCG-3′; and Hu-IL10-F: 5′-AGAACCTGAAGACCCTCAGGC -3′ and Hu-IL10-R: 5′-CCACGGCCTTGCTCTTGTT-3′. Amplicon specificity was carefully verified by the presence of a single melting temperature peak in the dissociation curves run after real-time RT-PCR, and the detection of a single band of the expected size was examined by electrophoresis. Real-time quantitative RT-PCR (qRT-PCR) was performed via the Applied Biosystems StepOne^TM^ detection system (Applied Biosystems) using the GoTaq® qPCR Master Mix (Promega Corp., Madison, WI, USA). All qRT-PCR experiments were performed at least 3 times. qRT-PCR data from the experiments were normalized using GAPDH as an endogenous control. All expression ratios were computed via the analysis of the relative gene expression using the ΔΔCt method through StepOne software version 2.0 (Applied Biosystems).

### Luciferase assays

RAW 264.7 cells (1 × 10^5^ cells) were plated in 48-well polystyrene plates and transfected with 1 μg of reporter plasmids using the Lipofectamine 2000 reagent (Invitrogen, Carlsbad, CA, USA). THP-1 cells (2 × 10^6^) were transfected with 500 ng of luciferase reporter plasmids using Nucleofector^TM^ Technology (Lonza, Basel, Switzerland) according to the manufacturer’s instructions. The PKR-Luc promoter plasmid used in the assays was kindly provided by Dr. Charles E. Samuel (University of California, Santa Barbara, USA). For the measurement of NF-kB transcriptional activity, the p6kB-Luc construct was used in our assays (kindly provided by Dr. Patrick Baeuerle). The iNOS promoter luciferase reporter plasmids were provided by Dr. David Geller of the University of Pittsburgh (PA, USA). After infection and treatment, the cells were washed with PBS, lysed according to the Dual Luciferase System protocol (Promega), and analyzed in the GloMax®-Multi (Promega Corp., Madison, WI, USA). The plasmid pBlue3’LTR-luc was provided by Dr. Reink Jeeninga and Dr. Ben Berkhout through the NIH AIDS Reagent Program (Division of AIDS, NIAID, NIH)^22^,^23^.

### Plasmids for Tat overexpression

Cloned plasmids with the Tat gene isoforms were kindly provided by Dr. Mauro Giacca (ICGEB - International Centre for Genetic Engineering and Biotechnology, Italy). The group provided two isoforms: Tat-86 wild-type and Tat86 R(49–57)A, which is a basic-domain mutant^19^. The sequences were amplified by PCR and subcloned into pEGFP-N2 (ClonTech) between the HindIII and EcoRI restriction sites. The constructs were transfected into THP-1 cells (2 × 10^6^) using 500 ng of DNA with the Nucleofector^TM^ Technology (Lonza, Basel, Switzerland) according to the manufacturer’s instructions.

### Statistical analyses

Data were analyzed by One-way ANOVA for independent samples using Prism 5 software. Data are expressed as the average of three independent determinations, and significant differences are indicated by p < 0.05.

### Ethics Statement

The methods carried out in this work are in accordance with the guidelines approved by the Ethical Committee of biological Research Experimentation, Federal University of Rio de Janeiro, Brazil. All the experimental protocols were approved by Oswaldo Cruz Foundation/Fiocruz Research Ethics Committee (Rio de Janeiro, RJ, Brazil), under the number 397–07 and Federal University of Rio de Janeiro Committee for Animal Use (permit numbers: IMPPG 024 and IBCCF171.

## Results

### Tat enhances PKR expression in macrophages infected with *L. amazonensis*

**Figure 1:**
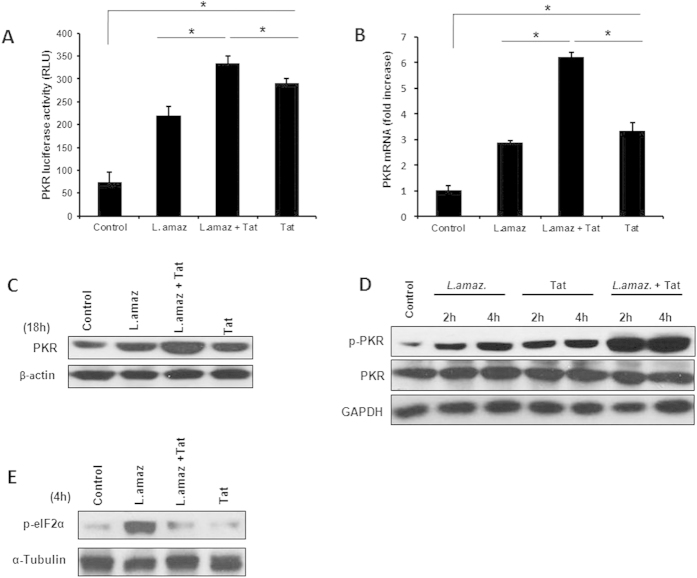
HIV-Tat protein induces PKR expression in infected macrophages. (**A**) RAW 264.7 cells were transiently transfected with a PKR-promoter-luciferase reporter plasmid, then treated with Tat (100 ng/mL) and/or infected with *L. amazonensis* 24 h after transfection. Whole-cell lysates were analyzed for luciferase activity 24 hours later. (**B**) THP-1 cells were infected with *L. amazonensis* for one hour, non-internalized promastigotes were washed out, fresh medium was added and then the cells were treated for an additional three hours with Tat (100 ng/mL). Total RNA was extracted and a quantitative real-time RT-PCR was performed. Western blot was performed using anti-PKR (**C**), anti-phospho-PKR (**D**) or anti-phospho-eIF2α (**E**) antibodies in infected and/or treated THP-1 cells as indicated. The results are representative of three independent experiments. *P < 0.05.

*L. amazonensis* induces the expression of PKR and triggers PKR signaling in macrophages^6^,^10^. Because PKR signaling plays a pivotal role in the host macrophage-*Leishmania* interaction, we investigated whether the HIV-1 Tat protein would enhance the expression and the levels of activated PKR in *Leishmania*-infected macrophages. We used a luciferase gene reporter construct containing inducible elements of the PKR promoter to test the role of Tat in *Leishmania-*infected macrophages. As observed in Fig. 1A, the addition of Tat increased the activity of the PKR promoter and enhanced the luciferase levels induced by parasite infection. Accordingly, PKR expression was highly increased in infected macrophages treated with Tat (Fig. 1B,C). The pre-incubation of HIV-1 Tat with *Leishmania* did not affect the parasite association with macrophages or the parasite load 48 hours post-infection, thereby ruling out a direct effect of Tat on the parasite (Supplementary Figure 1). Importantly, PKR underwent an increase in phosphorylation in *Leishmania*-infected cells treated with Tat (Fig. 1D), as revealed by the use of anti-pPKR (Thr451). Therefore, we investigated whether the main PKR substrate, the initiation factor eIF2α, was phosphorylated. Figure 1E shows that eIF2α was phosphorylated in infected macrophages. However, the addition of Tat remarkably reduced eIF2α phosphorylation, suggesting that Tat may be a PKR substrate and compete with eIF2α, as described elsewhere^14^. Taken together, these results indicate that HIV-1Tat is able to induce an increase in PKR levels and phosphorylation upon *Leishmania* infection.

### PKR is critical for the Tat-induced enhancement of intracellular *Leishmania* growth

**Figure 2:**
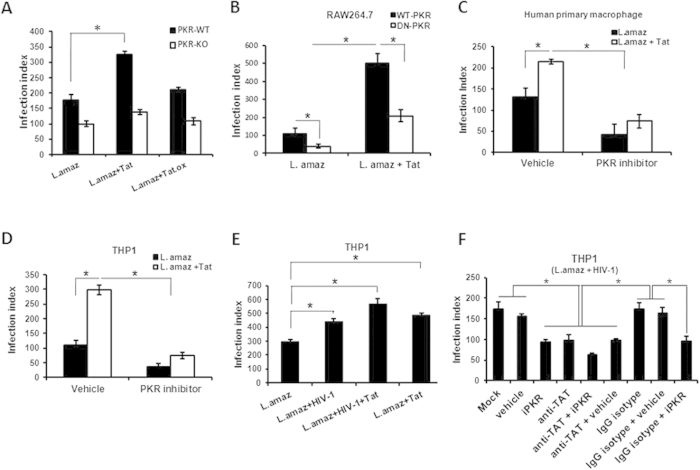
HIV-1 Tat protein induces *Leishmania* multiplication in macrophages in a PKR-dependent manner. The infection index was measured in (**A**) Peritoneal macrophages (wild-type or PKR knockout) and (**B**) RAW 264.7 cells stably transfected with either the empty vector (RAW-WT-Bla PKR cells) or the dominant-negative PKR K296R (RAW-DN-PKR cells) infected with the promastigote forms of *L. amazonensis* for 24 hours, then treated with Tat (100 ng/mL) for an additional 48 hours. (**C**) Human primary macrophages and (**D**) THP-1 cells were also infected with *L. amazonensis* and treated with Tat (100 ng/mL) and 300 nM of the PKR inhibitor prior to amastigote quantification. (**E**) THP-1 cells were infected with HIV-1 and *L. amazonensis* promastigotes and treated with recombinant Tat (100 ng/mL). (**F**) Alternatively, the co-infection was treated with anti-Tat serum plus PKR inhibitor. The results are representative of three independent experiments. *P < 0.05.

The above results prompted us to investigate the impact of the Tat-mediated enhancement of PKR activation on *Leishmania* intracellular growth. To this end, we used a RAW 264.7 cell line stably expressing a dominant-negative (DN)-PKR protein^24^ or PKR-KO macrophages. As depicted in Fig. 2A,B, Tat-induced growth of *Leishmania* was impaired in PKR-KO macrophages and DN-PKR RAW 264.7 cell line, thus suggesting that PKR is crucial for the Tat effect on intracellular parasite replication. To corroborate these results, we treated the *Leishmania*-infected THP-1 macrophage cell line or primary human macrophages with the PKR inhibitor CAS 608512-97-6 in the presence of Tat. Accordingly, PKR inhibition strongly reduced the effect of Tat on parasite growth (Fig. 2C,D). Moreover, the pre-incubation of iPKR with *Leishmania* did not affect the parasite association with macrophages or the parasite load 48 hours post-infection, thereby ruling out a direct effect of iPKR on the parasite (Supplementary Figure 1). Next, we addressed the effect of Tat in the context of HIV-1 and *Leishmania* co-infection. As expected, HIV-1 infection stimulated parasite growth, and additional treatment with Tat almost doubled parasite replication (Fig. 2E). Then, we investigated whether cells exposed to both HIV-1 and *L. amazonensis* exhibited enhanced parasite growth in a TAT/PKR-dependent manner. Figure 2F shows that PKR inhibition reduced parasite growth in HIV-1-/*Leishmania* co-infected cells. The same effect was observed when neutralizing anti-Tat antibody was added to the cultures. Moreover, the parasite load was further reduced when human macrophages were treated with iPKR and anti-Tat was added to the infected cells. Taken together, this set of results strongly suggests that Tat requires host cell PKR signaling to promote the growth of amastigotes in macrophages.

### *L. amazonensis* infection down regulates Tat-induced NF-κB activation downstream of PKR signaling

**Figure 3:**
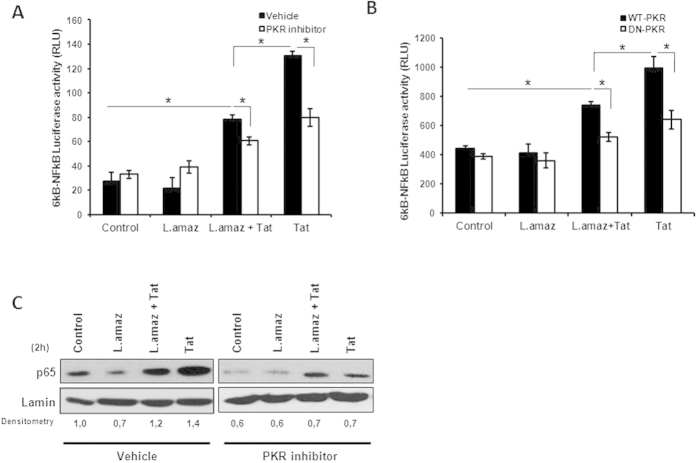
*L. amazonensis* and Tat induced NF-kB activation downstream of PKR signaling. (**A**) THP-1 and (**B**) RAW 264.7 cells stably transfected with either the empty vector (RAW-WT-Bla PKR cells) or the dominant-negative PKR K296R (RAW-DN-PKR cells) were transiently transfected with the 6kB-Luciferase consensus vector. Then, 24 h post-transfection the cells were infected with *L. amazonensis* for 18 h and/or treated with Tat (100 ng/mL). After twenty-four hours, whole-cell lysates were analyzed for luciferase activity. (**C**) Western blot was performed for THP-1 cell nuclear extracts. The cells were treated with Tat and/or PKR two hours post-infection. Nuclear proteins were extracted and incubated with the anti-p65 antibody in PVDF membranes. The results are representative of three independent experiments. *P < 0.05.

The transcription factor NF-κB is activated in viral and parasitic infections^25^. Briefly, the heterodimer RelA/p50 is translocated to the nuclei of infected cells and regulates the expression of a number of cytokines^26^. RelA/p50 is also important for HIV-1 LTR expression and may be induced by bacterial co-infections^27^ or by HIV-1-encoded proteins such as Tat^28^. We investigated the activation of NF-κB in *L. amazonensis*-infected macrophages treated with Tat and the PKR inhibitor through gene reporter assays (Fig. 3A). The NF-κB reporter construct was induced by Tat, and the inhibition of PKR partially prevented this effect. Importantly, Tat treatment increased NF-κB activation in uninfected cells, while *Leishmania* infection diminished this activation. These results were corroborated by employing the DN-PKR-RAW 264.7 cell line, thereby strengthening the hypothesis that Tat-mediated NF-κB activation was dependent on PKR signaling and that *Leishmania* infection reduced this effect (Fig. 3B). Next, we investigated the nuclear levels of RelA (p65) induced by Tat and the dependence of this phenomenon on PKR. Figure 3C shows that Tat increased the nuclear levels of RelA and that PKR inhibition prevented this effect. Taken together, these observations corroborate previous results on the role of Tat in NF-κB activation^29^ and demonstrate the importance of PKR signaling in this event. Importantly, *L. amazonensis* infection *per se* did not induce the canonical NF-κB p65/50 dimer, as previously described^30^.

### *Leishmania* infection reduces PKR-dependent Tat-induced iNOS expression

**Figure 4:**
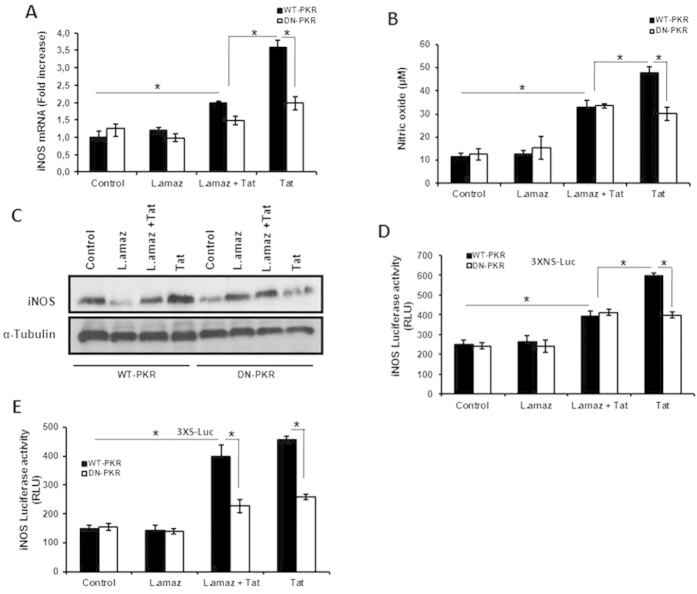
*L. amazonensis* reduces iNOS expression levels induced by treatment with Tat in a PKR-dependent manner. (**A**) RAW 264.7 cells stably transfected with either the empty vector (RAW-WT-Bla PKR) or the dominant-negative PKR K296R (RAW-DN-PKR) were infected with *L. amazonensis*. Non-internalized promastigotes were washed out and fresh medium was added, and then the cultures were treated for an additional three hours with Tat (100 ng/mL). Total RNA was extracted and a quantitative real-time RT-PCR for iNOS was performed. (**B**) RAW 264.7 cell lines were infected with *L. amazonensis* and/or treated with Tat (100 ng/mL) for 24 h. Then, the supernatant was analyzed for the presence of nitric oxide through the Griess reaction. (**C**) RAW 264.7 cell lines were infected for one hour with *L. amazonensis*. Non-internalized promastigotes were washed out and fresh medium was added, and then the cultures were treated for an additional five hours with Tat (100 ng/mL). Western blot analysis of iNOS protein expression was performed. RAW 264.7 cell lines were transiently transfected with the 3XNS-Luc (**D**) or 3XS-Luc (**E**) vectors. Then, 24 h post-transfection the cells were infected with *L. amazonensis* for 18 h and/or treated with Tat (100 ng/mL). After twenty-four hours, the whole cell lysates were analyzed for luciferase activity. The results are representative of three independent experiments. *P < 0.05.

The production of nitric oxide (NO) is associated with the activation of macrophages and can reduce the number of *Leishmania* amastigotes^31^. Because HIV-1 Tat has been demonstrated to induce iNOS^32^, we investigated whether *L. amazonensis* interferes with iNOS expression and NO production in the context of the Tat-PKR axis. We found that Tat induced iNOS expression in a PKR-dependent fashion (Fig. 4A). The presence of *Leishmania* reduced this effect, suggesting that *Leishmania* impaired the induction of iNOS by Tat. Accordingly, NO production followed the same pattern (Fig. 4B). These results were corroborated by immunoblot assays. Figure 4C clearly shows that Tat induced iNOS expression via PKR and that this effect was reduced in the presence of the parasite. To evaluate the effect of Tat on the NF-κB/STAT1 regulatory elements present in the iNOS promoter^33^, we performed luciferase reporter assays. Sole infection by *L. amazonensis* did not induce the constructs carrying either the NF-κB/STAT1 or STAT1 regulatory element (Fig. 4D,E, respectively). Importantly, Tat treatment resulted in the activation of both reporter constructs, but *Leishmania* infection reduced this effect on the NF-κB/STAT1 construct. Based on these results, we suggest that *L. amazonensis* reduced the expression of iNOS induced by HIV1 Tat.

### The Tat basic domain (49–57) is required for PKR activation and parasite growth enhancement in macrophages

**Figure 5:**
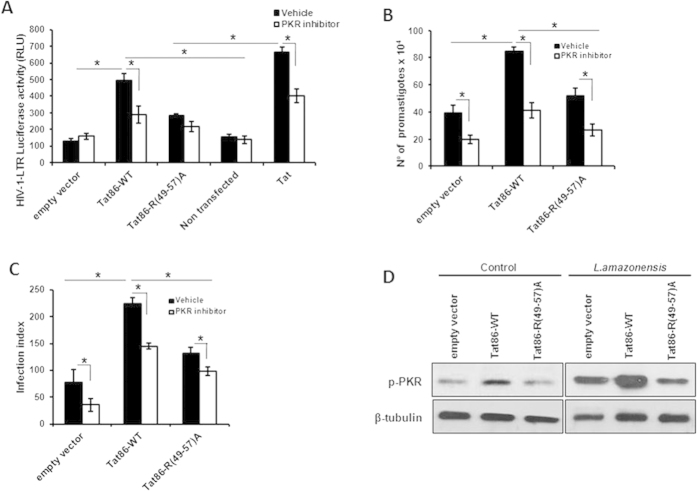
The basic domain of Tat is required for PKR activation in *L. amazonensis*-infected macrophages. THP-1 cells were transiently transfected with the empty vector, Tat86-WT or Tat86 R(49–57)A plasmids as previously described. Twenty-four hours post-transfection, the cells were differentiated into macrophages with PMA treatment for 4 days. (**A**) The cells were co-transfected with the LTR-Luciferase vector and treated with or without PKR inhibitor and/or Tat (100 ng/mL). Whole-cell lysates were analyzed for luciferase activity 24 hours later. (**B**) The cells were infected with *L. amazonensis* for 48 h, and the number of parasites that emerged from the infected cells was counted in the supernatants. (**C**) Alternatively, the intracellular infection index was measured. (**D**) Western blotting was performed for THP-1 transfected cells infected with *L. amazonensis* with anti-phospho-PKR at the indicated time points. The results are representative of three independent experiments. *P < 0.05.

Tat binds to PKR through a cognate basic domain (49–57 aa)^16^. To address whether the Tat effect on the modulation of parasite infection requires the Tat-basic domain, we used a Tat mutant construct with the arginine (R) residues replaced by alanine (A) (Tat86-R(49–57)A) to evaluate whether both Tat constructs could activate the HIV-1-LTR reporter construct in THP-1 macrophages. As predicted, Tat86-R (49–57)A could not induce HIV-1 LTR activity (Fig. 5A), while Tat86-WT strongly stimulated the LTR regulatory elements. Importantly, the pharmacological inhibition of PKR decreased LTR induction. These results prompted us to evaluate the effect of Tat86-R(49–57)A on intracellular parasite growth. As shown in Fig. 5B,C, Tat86-R(49–57)A did not promote the enhancement of the parasite load. Then, we analyzed the role of Tat in PKR activation in macrophages infected with *Leishmania* infection. As observed in Fig. 5D, the expression of Tat86-R(49–57)A in *Leishmania*-infected macrophages did not activate PKR, while the Tat86-WT isoform potentiated pPKR levels. Taken together, our results indicate that Tat favors *L. amazonensis* growth in a manner that is dependent on the Tat basic region and is possibly mediated through PKR.

### Tat favors *L. amazonensis* growth through PKR-dependent IL-10 expression

**Figure 6:**
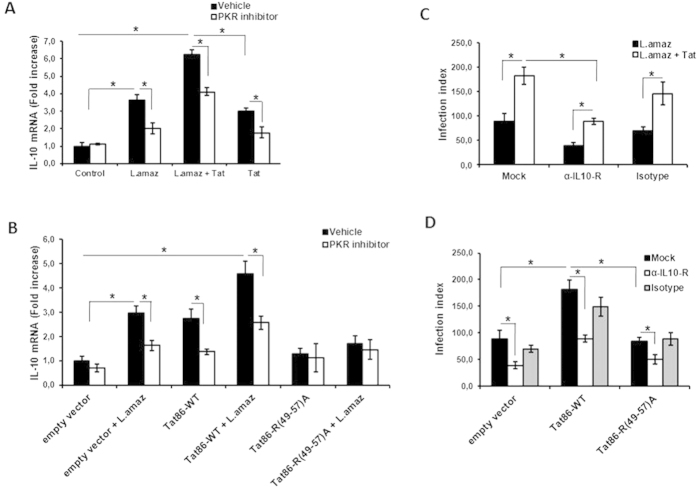
IL-10 signaling is necessary for *Leishmania* growth induced by PKR activation via Tat. THP-1 cells were treated with Tat (**A**) or transfected with Tat86-WT or Tat86-R(49-57)R (**B**) and then infected with *L. amazonensis* for 4 h. Total RNA was extracted and analyzed for IL-10 transcripts using quantitative real-time PCR. The infection index was evaluated for THP-1 infected cells treated with neutralizing anti-IL-10 R antibody or the IgG isotype (**C**) and in THP-1 cells transfected with Tat86-WT or Tat86-R(49-57)R (**D**). The results are representative of three independent experiments. *P < 0.05.

We previously reported that PKR activation induces IL-10, which in turn favors *L. amazonensis* infection^6^. Because HIV-1 Tat may also induce IL-10 expression, we studied the expression of IL-10 in infected macrophages treated with Tat and tested the role of PKR on IL-10 induction. Figure 6A shows that Tat potentiates the ability of *L. amazonensis* to induce IL-10 expression in macrophages in a PKR-dependent fashion. Similar results were obtained in infected DN-PKR RAW 264.7 macrophages (Supplementary Figure 2). Next, we tested the requirement for the Tat basic domain for IL-10 induction in infected macrophages. Our results showed that the Tat86 construction with the mutated basic domain was unable to induce IL-10 and, as predicted, it could not synergize with the *L. amazonensis* infection (Fig. 6B). To further characterize the effect of the axis formed by Tat/IL-10 on the exacerbation of parasite growth, we assessed the parasite growth in infected cells treated with Tat in the presence of an anti-IL-10 receptor neutralizing antibody. Blockage of the IL-10 receptor abolished the effect of Tat on parasite growth (Fig. 6C). Moreover, the enhancement of *Leishmania* growth by Tat86-WT was also dependent on IL-10, whereas the mutant construct Tat86-R (49–57)A could not exacerbate parasite growth (Fig. 6D).

## Discussion

The soluble HIV-1 Tat protein can be found in in the serum of HIV-1 infected patients as well as in the tissues^34^. Once in the extracellular milieu, Tat can be taken up by bystander host cells^35^. Inside the cells, Tat is able to interact and activate signaling pathways^36^, which can result in the induction of cytokines such as IL-10 13 and TGF-β^37^. Tat was previously reported to induce the uptake of *Leishmania* parasites^38^. We also demonstrated that Tat increased the intracellular growth of *Leishmania amazonensis* and revealed the importance of secreted factors in the *Leishmania*-exacerbation effect mediated by Tat^21^. However, neutralization of two soluble factors (Prostaglandin E2 and TGF-β) did not completely abolish the up-modulating effect of Tat on *Leishmania*^21^, suggesting that other mediators (or even interference with a cellular signaling pathway) could participate together with these secreted molecules. The dsRNA-activated kinase PKR can be targeted and activated by Tat^14^,^17^. Importantly, previous work demonstrated a pivotal role for PKR in the growth of intracellular parasites^6^,^10^,^11^. These observations strengthened our hypothesis that Tat could also affect the cell signaling cascade without requiring prior cytokine secretion. In this work, we tested the hypothesis that Tat could modulate the growth of intracellular parasites in infected macrophages via PKR signaling and IL-10 expression.

Our results clearly showed that Tat potentiated PKR activation resulting from *Leishmania* infection. The PKR binding sequence of the main PKR substrate (the translation initiation factor eIF2α) shares amino acid sequence similarities with the phosphorylation region of HIV-1 Tat. Our results confirmed that *L. amazonensis* infection leads to PKR activation and the phosphorylation of eIF2α. This effect was prevented by the addition of HIV-1 Tat. HIV-1Tat 86 can be phosphorylated by PKR, thus competing with the PKR substrate eIF2α^14^. Importantly, the PKR promoter construct was activated by Tat treatment or *Leishmania* infection alone, and the association of both conditions increased the stimulation of the PKR promoter, probably through the induction of ISRE elements present in the construct. We cannot rule out a direct effect of HIV-1 Tat on the activation of these responsive elements. However, we observed the induction of IFN1β expression in Tat-treated macrophages (data not shown), which could lead to the activation of the PKR promoter.

As expected, based on our previous studies^21^, HIV-1 Tat enhanced amastigote growth in macrophages and now we show that this phenomenon requires PKR signaling. Importantly, the neutralization of HIV-1 Tat produced by HIV-1-infected macrophages reduced the parasite load, and this effect was even more accentuated when anti-HIV-1 Tat was added to iPKR-treated *Leishmania*-infected macrophages. PKR and Tat act together to induce IL-10 expression^39^ and our results reveal an important aspect of *Leishmania* and HIV-1 co-infection: HIV-1 Tat protein is able to induce IL-10 secretion in *Leishmania*-infected macrophages through PKR activation. This conclusion is supported by our results showing that IL-10 expression was highly induced in Tat-treated *Leishmania*-infected macrophages, while there was reduced expression of IL-10 in the infected DN-PKR RAW 264.7 cell line and human macrophages treated with iPKR. The blockage of the IL-10 receptor completely abrogated the Tat effect in promoting the intracellular growth of *Leishmania*, thus corroborating the notion that IL-10 is an essential effector of the Tat-PKR axis during co-infection. Moreover, we expressed in infected macrophages a Tat mutant construct in which the basic region was altered, which resulted in a protein that was unable to interact with PKR^19^. The expression of Tat86-R (49–57)A in infected macrophages did not enhance parasite growth or up-modulate IL-10 expression and PKR activation. We have not pursued co-immunoprecipitation studies to verify the direct binding of HIV-1 Tat 86 to PKR. However, our results are in accordance with previous reports that showed and mapped the minimum sequences involved in the direct interaction of Tat with PKR.

Considering the cascade of signals, HIV-1 Tat could induce IL-10 production even from non-infected cells, and this secreted IL-10 could act in nearby *Leishmania*-infected cells^40^. HIV-1 Tat can activate the canonical NF-κB pathway, leading to the nuclear translocation of the RelA/p50 heterodimer^41^, which may contribute to the expression of pro-inflammatory cytokines and iNOS. Our results show that Tat indeed promotes iNOS expression and NO production. However, both effects were reduced in *Leishmania*-infected macrophages. Accordingly, *L. amazonensis* subverts canonical NF-κB activation in macrophages and promotes the formation of the repressor homodimer p50/50 30, which silences the *nk* regulatory elements observed in the iNOS promoter. These results show that *L. amazonensis* interferes with cell host signaling induced by HIV-1 Tat, thereby reducing iNOS production and modulating NF-κB dimer activation. Taken together, we envisage a scenario in which released Tat enters the cell, interacts with PKR, and regulates its activation. This complex promotes nuclear translocation of NF-κB and IL-10 induction. Despite the fact that PKR activation could induce a harmful environment for *Leishmania* survival, the presence of the parasite overrides the effect of PKR action on iNOS expression, thereby permitting parasite survival and growth. Altogether, our data support the notion that Tat plays a pivotal role in favoring *L. amazonensis* infection through PKR signaling and IL-10 expression. Our results unveil the basic molecular mechanisms by which *Leishmania-*HIV-1 co-infection may result in the clinical aggravation of leishmaniasis symptoms, and may contribute to the development of novel treatment procedures.

## Additional Information

**How to cite this article**: Vivarini, C. *et al.* HIV-1 Tat protein enhances the intracellular growth of *Leishmania amazonensis* via the ds-RNA induced protein PKR. *Sci. Rep.* **5**, 16777; doi: 10.1038/srep16777 (2015).

## Supplementary Material

Supplementary Information
