# Supplementary material for: HIV-1 Tat protein enhances the intracellular growth of Leishmania amazonensis via the ds-RNA induced protein PKR
Source: Sci Rep. 2015 Nov 26;5:16777. doi: 10.1038/srep16777 (PMC4660360; doi:10.1038/srep16777)
Supplement: Supplementary Information [file srep16777-s1.doc]

**Supplemental Information**

**HIV-1 Tat protein enhances the intracellular growth of *Leishmania amazonensis* via the ds-RNA induced protein PKR**

**Áislan de Carvalho Vivarini*****, Renata de Meirelles Santos Pereira*****, Victor Barreto-de-Souza**‡**, Jairo Ramos Temerozo**‡**, Deivid C. Soares**†**, Elvira M. Saraiva**†**, Dumith Chequer Bou-Habib**^1^‡ **and Ulisses Gazos Lopes***^1^**.**

**Supplementary Figures**

**
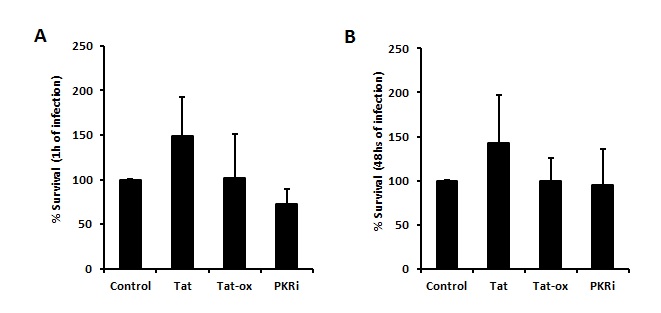
**

**Supplementary Figure 1 | Safety of HIV-1 Tat and the PKR inhibitor for *Leishmania* *amazonensis.*** Promastigotes were treated with 100 ng/mL HIV-1 Tat, oxidized-Tat [Tat-ox, inactive Tat], and 300 nM iPKR. The parasites were washed out after one hour and allowed to interact with peritoneal macrophages for (A) 1 h and (B) 48 h. Parasite-macrophage interactions were counted in Giemsa-stained cultures. The results are representative of three independent experiments and are shown as percentage of parasite association and parasite load. * P < 0.05.

**
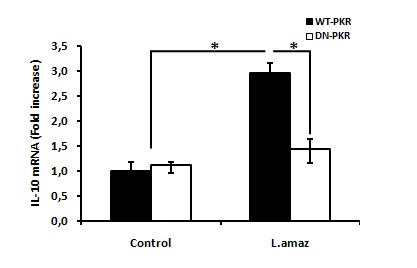
**

**Supplementary Figure 2 | PKR signaling is essential for augmentation of IL-10 transcripts due to *L. amazonensis* infection and HIV-1 Tat treatment.** (A) RAW 264.7 WT-PKR and DN-PKR cells were infected with *L. amazonensis* for one hour. Then, noninternalized promastigotes were washed out, fresh medium was added and the cells were treated for an additional three hours with Tat (100 ng/mL). Total RNA was extracted and analyzed for IL-10 transcripts using a quantitative real-time PCR. The results are representative of three independent experiments. *P < 0.05.
